# Impact of Myopia on the Utility of the Photopic Negative Response Ratio for Glaucoma Assessment

**DOI:** 10.3390/jcm14030682

**Published:** 2025-01-21

**Authors:** Young Gun Park, Chan Kee Park, Kyoung In Jung

**Affiliations:** Department of Ophthalmology, Seoul St. Mary’s Hospital, College of Medicine, The Catholic University of Korea, Seoul 06591, Republic of Korea; cuteyg2000@catholic.ac.kr (Y.G.P.); ckpark@catholic.ac.kr (C.K.P.)

**Keywords:** glaucoma, myopia, electroretinogram, photopic negative response

## Abstract

**Background**: The objective assessment of visual function is crucial in glaucoma management, highlighting the importance of electroretinography (ERG). This study investigates the diagnostic performance of photopic negative response (PhNR) amplitude and the normalized PhNR/b-wave ratio in diagnosing glaucoma, focusing on the impact of myopia. **Methods**: Ninety-one glaucoma patients and 19 glaucoma suspects were included, defining myopia as axial length (AL) > 24 mm or > 25 mm. Full-field photopic ERG used a red stimulus on a blue background. **Results**: Myopic glaucoma patients showed a higher PhNR/b-wave ratio than non-myopic patients (*p* = 0.023). AL negatively correlated with b-wave amplitude (r = −0.239, *p* = 0.012). PhNR amplitude demonstrated an area under the receiver operating characteristic curve (AUC) of 0.661 (*p* = 0.028) overall and was less effective in the myopic subgroup (AUC = 0.574, *p* = 0.082). The diagnostic performance of the PhNR/b-wave ratio did not achieve statistical significance in either the total group (AUC = 0.616, *p* = 0.114) or the myopic subgroup (AUC = 0.574, *p* = 0.332). **Conclusions**: Standardization using the PhNR/b-wave ratio did not enhance diagnostic accuracy over PhNR amplitude, particularly in myopic patients, underscoring the need for careful interpretation in myopia and further research to optimize electrophysiological diagnostics.

## 1. Introduction

Glaucoma, a leading cause of blindness, leads to the selective and progressive loss of retinal ganglion cells (RGCs) [1]. Myopia is recognized as a significant risk factor for glaucoma [2]. However, diagnosing glaucoma in individuals with myopia presents challenges due to the anatomical changes associated with increased axial length (AL) and ocular stretching, such as a thinner retinal nerve fiber layer (RNFL) and alterations in the optic disc and peripapillary area, including tilted optic discs and parapapillary atrophy [2,3]. Evaluating visual function in patients with glaucoma is fundamental for both diagnosing and monitoring glaucoma progression, and it can be even more critical in patients with high myopia due to these diagnostic challenges based on structural evaluation. Standard automated perimetry 24-2, the most common functional test for glaucoma, detects functional loss of RGCs only after 25–35% have been lost [4]. Moreover, visual field (VF) testing is subjective, and its accuracy is limited by factors such as fatigue, learning effects, and cognitive demands, as perception is required to respond to light stimuli during testing [5,6].

Objective electrophysiological tests, including pattern electroretinography (ERG) and photopic negative response (PhNR) measurement, offer potential advantages in detecting early-stage RGC dysfunction and can serve as complementary tests to VF assessments [7,8]. The PhNR, a negative wave that is detected after the b-wave in photopic full-field ERGs, is thought to originate from the spiking activity of RGCs [9]. Unlike pattern ERG, which requires refractive correction and good fixation, PhNR measurement is relatively independent of refractive errors, steady fixation control, and optical clarity [6,10]. Our previous research demonstrated that PhNR analysis performed well in the inter-eye structure–function relationship in glaucoma patients, especially at the early stage [11].

Research indicates that PhNR amplitude may be influenced by age, sex, and peripapillary RNFL thickness in healthy individuals [12,13]. Although the N95 amplitudes of pattern ERG responses decrease with early glaucomatous damage and are further reduced with increasing myopia [14,15], the effects of AL on PhNR in full-field ERG remain unexplored. Normalizing the PhNR amplitude by dividing it by the amplitude of the b-wave, referred to as the PhNR/b-wave ratio, has been suggested to reduce inter-individual variability, as amplitude can be affected by electrode location and individual characteristics, such as eye size [16,17,18]. However, it is known that b-wave amplitude decreases with increased AL [14,19]. The relationship between AL and b-wave amplitude may affect the diagnostic performance of the PhNR/b-wave ratio in distinguishing glaucoma in myopic patients.

To date, no published studies have thoroughly investigated the association between PhNR amplitude or the PhNR/b-wave ratio and AL. Therefore, this study aimed to examine the relationship between PhNR amplitude or the PhNR/b-wave ratio and AL and to assess whether the diagnostic ability of these measures is compromised in glaucoma patients with myopia, with a focus on the impact of myopia as an influencing factor rather than a causative one.

## 2. Materials and Methods

### 2.1. Study Design and Inclusion Criteria

This cross-sectional study received institutional review board approval from The Catholic University of Korea, Seoul, Republic of Korea, and adhered to the principles of the Declaration of Helsinki. Informed consent was obtained from all participants prior to enrollment. Patients meeting specific inclusion criteria for primary open angle glaucoma and glaucoma suspect were recruited from the glaucoma clinic at Seoul St. Mary’s Hospital between December 2016 and June 2017. Eligible participants had a best-corrected visual acuity of 20/30 or better, open-angle configuration, and an AL of <30 mm. Exclusion criteria included any history of uveitis; neurologic conditions affecting vision; or retinal disorders, such as vein or artery occlusion, macular disease, or prior retinal surgery. Patients with advanced glaucoma, defined by a mean deviation (MD) worse than −20 dB, or those who had undergone intraocular surgery ≤ 6 months ago were also excluded.

### 2.2. Clinical Evaluation and Diagnosis

Participants underwent a comprehensive ophthalmic examination, including slit-lamp biomicroscopy, Goldmann applanation tonometry, and measurements of central corneal thickness and AL using ocular biometry (IOL Master 500; Carl Zeiss Meditec, Inc., Dublin, CA, USA). Gonioscopic evaluation, red-free fundus photography, and stereoscopic optic disc photography were also performed.

Glaucoma was diagnosed based on the presence of glaucomatous optic disc changes, such as diffuse or focal rim thinning, notching, or RNFL defects coupled with corresponding VF abnormalities. Eyes were classified as glaucoma suspects if they exhibited glaucomatous structural changes (e.g., optic disc rim thinning, notching, or RNFL defects) in the absence of VF defects that met the criteria for glaucomatous VF loss.

#### 2.2.1. Optical Coherence Tomography

Peripapillary RNFL thickness was measured using Cirrus spectral-domain optical coherence tomography (SD-OCT) (version 6.0; Carl Zeiss Meditec, Inc.) with the Optic Disc Cube 200 × 200 scan mode. Ganglion cell inner plexiform layer (GCIPL) thickness was evaluated using the macular cube scan with GCA software [20,21]. Average peripapillary RNFL thickness, as well as both the average and minimum GCIPL thickness (the thinnest GCIPL thickness over a single meridian crossing the annulus), were recorded. Images with a signal length of <6 and with evident misalignment or segmentation errors were excluded from the analysis.

#### 2.2.2. Visual Field Testing

All participants underwent standard automated perimetry using the 24-2 test pattern on a Humphrey field analyzer (Carl Zeiss Meditec, Inc.) using the Swedish interactive threshold algorithm standard strategy. VF defects were defined as a cluster of at least three contiguous points with reduced sensitivities at the 5% level, one of which must be at the 1% level, on the pattern deviation plot. Only reliable tests with <20% fixation losses, false positives, or false negatives were included.

#### 2.2.3. Full-Field Photopic ERG

**Figure 1:**
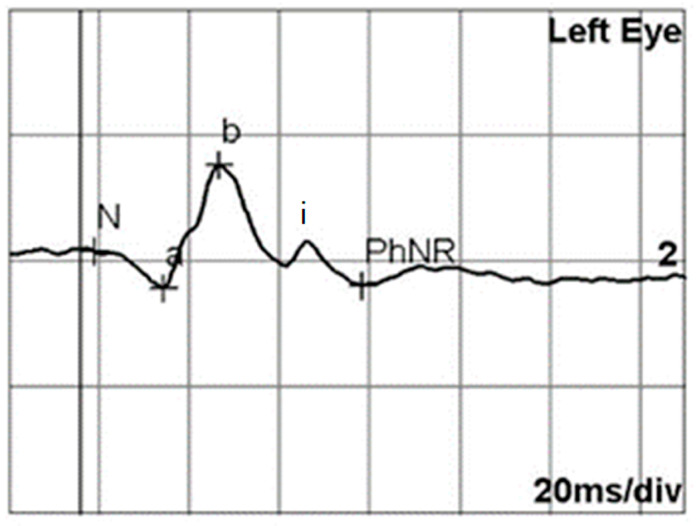
A representative electroretinogram recorded using a red stimulus on a blue background. The a-wave, b-wave, i-wave, and photopic negative response (PhNR) are labeled. The PhNR amplitude was measured from the baseline to the negative trough occurring approximately 80 ms after the b-wave and i-wave.

Full-field ERG was performed using the RETI-port system (Roland Consult, Brandenburg an der Havel, Germany). Corneal responses were recorded using Dawson–Trick–Litzkow electrodes, with reference electrodes positioned on the forehead and ground electrodes placed on the lateral canthi. Pupils were dilated with a combination of phenylephrine/tropicamide. After a 10-min period of light adaptation, the PhNR amplitude was recorded using a red stimuli of 1.6 cd s/m^2^ on a blue background of 25 cd/m^2^, following the guidelines set by the International Society for Clinical Electrophysiology [22]. The stimulus was presented at a frequency of 1.089 Hz with a duration of 4 ms. The signals were amplified (band-pass, 1–300 Hz). The a-wave amplitude was measured from the baseline to the first negative trough, whereas the b-wave amplitude was measured from the trough to the peak of the initial positive wave. The PhNR amplitude was calculated as the difference from the baseline to the negative trough occurring approximately 80 ms after the b-wave and i-wave (Figure 1). The i-wave was defined as the first positive deflection following the b-wave [23]. The PhNR/b-wave amplitude ratio was then calculated.

### 2.3. Statistical Analysis

Statistical analysis was conducted using SPSS for Windows version 23.0 (IBM Corporation, Armonk, NY, USA). Continuous variables were compared using Student’s *t* tests, whereas categorical variables were analyzed using the chi-squared test. Linear regression analyses were used to assess the relationships between clinical parameters and ERG measurements. The diagnostic performance of the parameters was evaluated by calculating the area under the receiver operating characteristic curve (AUC). Correction for multiple comparisons was not applied to minimize the risk of a type II error (false negative), as this study was designed as an exploratory investigation. A *p*-value < 0.05 was considered statistically significant.

## 3. Results

This study included 91 patients diagnosed with glaucoma and 19 subjects with suspected glaucoma. Glaucoma patients had thinner RNFLs and GCIPLs and worse VF results (lower MD and higher pattern standard deviation) than glaucoma suspects (all *p* < 0.05) (Table 1). The PhNR amplitude was lower in the glaucoma patients compared to the glaucoma suspects (*p* = 0.035). Most other parameters, such as age, sex, central corneal thickness, spherical equivalent, and AL, did not exhibit significant differences between the groups (all *p* > 0.05).

Subjects were categorized into non-myopia (*n* = 42; AL, <24 mm) and myopia (*n* = 68; AL, ≥24 mm) subgroups (Table 2). A statistically significant difference in age was observed, with the non-myopia group being older than the myopia group (*p* < 0.001). Gender distribution also varied significantly, with more men present in the myopia group (*p* < 0.001). The AL was significantly longer in the myopia group (25.6 ± 1.1 mm vs. 23.2 ± 0.6 mm, *p* < 0.001). When myopia was categorized based on an AL cutoff of 25 mm, the myopic group was significantly younger, with an average age of 44.8 ± 10.3 years (*p* < 0.001). The gender distribution differed significantly between the groups (*p* = 0.001). AL was significantly longer in the myopic group, averaging 26.2 ± 0.9 mm compared to 23.2 ± 0.86 mm in the non-myopic group (*p* < 0.001).

SD-OCT revealed a significant reduction in average RNFL thickness in the myopia group (74.5 ± 11.8 µm) compared to the non-myopia group (80.7 ± 10.6 µm, *p* = 0.006) (Table 3). Similarly, the average GCIPL thickness was significantly less in the myopia group (70.4 ± 9.0 µm) compared to the non-myopia group (75.4 ± 8.2 µm, *p* = 0.004). Regarding ERG parameters, there were no significant differences between the groups when myopia was categorized as AL > 24 mm. The PhNR/b-wave ratio was significantly greater in the myopic group (0.33 ± 0.12) compared to the non-myopic group (0.29 ± 0.09, *p* = 0.023) when myopia was categorized as AL > 25 mm.

The correlation analysis demonstrated a significant negative correlation between AL and b-wave amplitude (r = −0.230, *p* = 0.016) (Table 4) and a positive correlation between AL and the PhNR/b-wave ratio (r = 0.239, *p* = 0.012).

Among all patients, the multivariate regression analysis identified average GCIPL thickness as a significant factor influencing both PhNR amplitude and the PhNR/b-wave ratio, with a positive association (*p* = 0.001 for PhNR amplitude and *p* = 0.036 for the PhNR/b-wave ratio) (Table 5). In the non-myopia group, the average GCIPL thickness was significantly positively associated with both the PhNR amplitude (*p* = 0.006) and the PhNR/b-wave ratio (*p* = 0.004). In the myopia group, the PhNR amplitude was negatively associated with age (*p* = 0.008) and positively associated with average GCIPL thickness (*p* = 0.045). The PhNR/b-wave ratio was significantly associated with AL (*p* = 0.040), but the association between average GCIPL thickness and the PhNR/b-wave ratio was not significant (*p* = 0.681).

The receiver operating characteristic curve analysis showed that OCT parameters, particularly minimum GCIPL thickness, had the greatest diagnostic accuracy for distinguishing glaucoma patients from glaucoma suspects, with an AUC of 0.927 (*p* < 0.001) in the total population (Table 6). For the non-myopia group, the minimum GCIPL thickness achieved an AUC of 0.939 (*p* < 0.001), whereas, in the myopia group, it achieved an AUC of 0.921 (*p* < 0.001). The PhNR amplitude had an AUC of 0.661 (*p* = 0.028), whereas the PhNR/b-wave ratio did not achieve statistical significance in the total population (AUC = 0.616, *p* = 0.114). In the non-myopia group, the ERG parameters, particularly the PhNR amplitude, demonstrated an AUC of 0.715 (*p* = 0.011), and the PhNR/b-wave ratio obtained an AUC of 0.696 (*p* = 0.021) in distinguishing between glaucoma patients and suspects. In the myopic group, the ERG parameters, particularly the PhNR amplitude/b-wave ratio, showed limited diagnostic performance for distinguishing between glaucoma patients and suspects (AUC = 0.574, *p* = 0.332).

**Figure 2:**
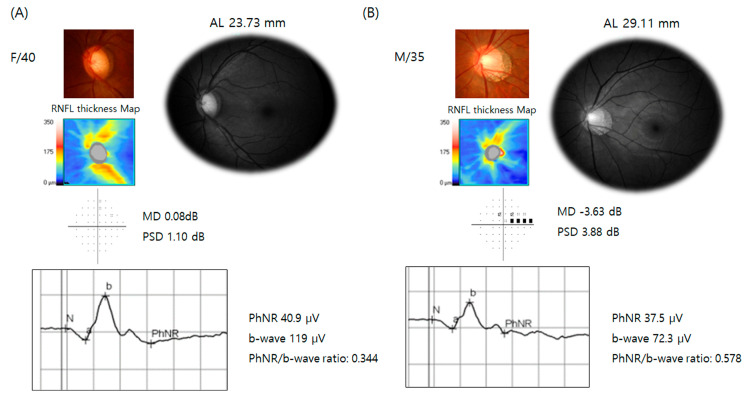
Representative cases of photopic negative response (PhNR) in suspected glaucoma without high myopia and early-stage glaucoma with high-myopia. (**A**) A 40-year-old female with suspected glaucoma with optic disc cupping and rim thinning. The axial length (AL) was 23.73 mm. The PhNR amplitude was 40.9 µV, the b-wave amplitude was 119 µV, and the PhNR/b-wave ratio was 0.344. (**B**) A 35-year-old male with high myopia (AL = 29.11 mm) and early-stage glaucoma (mean deviation [MD] = −3.63 dB). The PhNR amplitude was 37.5 µV, the b-wave amplitude was 72.3 µV, and the PhNR/b-wave ratio was 0.578, which is higher than in the glaucoma suspect case (**A**).

Representative cases are presented in Figure 2. The first case is a 40-year-old female patient with suspected glaucoma, who exhibited a high cup-to-disc ratio with inferior rim thinning and an AL of 23.73 mm. Her PhNR amplitude was 40.9 µV, her b-wave amplitude was 119 µV, and her PhNR/b-wave ratio was 0.344. The second case is a 35-year-old male patient with high myopia (AL = 29.11 mm) and early-stage glaucoma (MD = −3.63 dB). His PhNR amplitude was 37.5 µV, and his b-wave amplitude was 72.3 µV, resulting in a PhNR/b-wave ratio of 0.578, which is notably higher than that of the glaucoma suspect.

## 4. Discussion

This study demonstrated that glaucoma patients had lower PhNR amplitudes compared to glaucoma suspects (*p* = 0.035). Myopic glaucoma patients exhibited a greater PhNR/b-wave ratio compared to non-myopic glaucoma patients (*p* = 0.023). AL negatively correlated with b-wave amplitude (r = −0.023, *p* = 0.016) and positively correlated with the PhNR/b-wave ratio (r = 0.239, *p* = 0.012) but showed no significant relationship with PhNR amplitude (r = 0.032, *p* = 0.742). Among myopic patients, the PhNR amplitude was positively correlated with average GCIPL thickness, whereas the PhNR/b-wave ratio correlated positively with AL (*p* = 0.040) rather than GCIPL thickness (*p* = 0.681) in the multivariate analysis. The PhNR/b-wave ratio did not achieve statistical significance in the myopic patient group (AUC = 0.574, *p* = 0.332), whereas it did achieve statistical significance in the non-myopic patient group (AUC = 696, *p* = 0.021).

The PhNR is an ERG component that primarily reflects the functional integrity of the inner retinal layers, particularly the RGCs [6,10,24]. Given its slower timing, the PhNR is believed to involve contributions not only from the RGCs but also from amacrine and glial cells [24,25]. Our study shows that PhNR amplitude is decreased in glaucoma patients when compared to glaucoma suspects. The PhNR amplitude was significantly associated with average GCIPL thickness in patients with glaucoma or glaucoma suspects, regardless of AL, suggesting that it represents a reliable indicator of RGC dysfunction and loss. Previous studies have also demonstrated that the PhNR amplitude is reduced in pre-perimetric glaucoma, with further reductions in perimetric glaucoma and a significant positive correlation with average RNFL or GCIPL thickness, supporting our findings [11,18,26].

There is some overlap in PhNR amplitude between open-angle glaucoma and healthy eyes, which is greater than that observed for the ganglion cell complex measured by OCT [6]. To reduce inter-individual variability, normalization techniques such as the PhNR/b-wave ratio can be employed, which may mitigate variation due to electrode location and individual characteristics [18]. The PhNR/b-wave ratio is calculated by dividing the PhNR amplitude by the b-wave amplitude [16,17,18].

Regarding wave characteristics, there was no significant difference in a-wave or b-wave amplitudes when comparing glaucoma suspects and patients in our study. Although certain studies have observed a reduction in a-wave amplitude in glaucoma patients compared to control subjects, there is no conclusive evidence indicating that a reduced b-wave is characteristic of glaucoma [17,18,27,28].

This study also contended that AL negatively correlates with b-wave amplitude. The elongation and thinning of ocular structures in myopia are associated with significant structural and functional alterations in the retina, optic disc, and peripapillary area [14]. Multiple studies have documented a notable reduction in the b-wave amplitude under photopic and scotopic conditions in individuals with high myopia [14,29]. In a study of young healthy subjects aged 20–29 years, large PhNR amplitude was associated with a thicker peripapillary RNFL but not with AL, in line with the findings of our study [13]. Further, in the same study, the W ratio (b-wave voltage − PhNR voltage)/(b-wave voltage − a-wave voltage) was positively associated with AL in the multivariate analysis, corresponding with our study [13]. However, no study to date has investigated the effects of AL on the PhNR/b-wave ratio in glaucoma patients. Our study found that the PhNR/b-wave ratio was associated with average GCIPL thickness in all patients but not the myopia subgroup. In the myopia subgroup, a longer AL was associated with a greater PhNR/b-wave ratio. These findings suggest that, although the PhNR amplitude is unaffected by AL, the PhNR/b-wave ratio is influenced by a longer AL due to the impact of AL on b-wave amplitude. The positive correlation between AL and PhNR/b-wave ratio in myopic patients highlights the need for careful consideration of AL in the interpretation of the PhNR ratio.

In terms of diagnostic ability, the PhNR amplitude demonstrated discriminatory power for glaucoma, with an AUC of 0.715 (*p* = 0.011), and the PhNR/b-wave ratio showed an AUC of 0.696 (*p* = 0.021) in the non-myopic group. In the non-myopic group, the diagnostic ability for glaucoma was similar between the PhNR amplitude and the PhNR/b-wave ratio, aligning with previous studies [16,18]. Preiser et al. reported that the diagnostic ability of the PhNR/b-wave ratio (80.1%) for glaucoma was greater than that for the PhNR amplitude (77.9%) [18]. However, a direct statistical comparison of the diagnostic performance between the PhNR/b-wave ratio and the PhNR amplitude was not performed [18]. Kirkiewicz et al. found no significant differences in diagnostic ability between PhNR amplitude and the PhNR/b-wave ratio in glaucoma diagnosis [16].

The PhNR amplitude demonstrated discriminatory power for glaucoma, with an AUC of 0.661 (*p* = 0.028) in the total group, and showed marginal significance (AUC = 0.574, *p* = 0.082) in the myopic subgroup. The PhNR/b-wave ratio did not achieve statistical significance in the total patient group (AUC = 0.616, *p* = 0.114) or the myopic patient subgroup (AUC = 0.574, *p* = 0.332). These findings suggest that the diagnostic utility of the PhNR amplitude, especially the PhNR/b-wave ratio, may vary depending on myopia status. When adopting the PhNR/b-wave ratio for glaucoma diagnosis in myopic patients, caution is advised. Electrophysiological tests, such as ERG, are not yet widely adopted in clinical practice, partly due to the lack of detailed and diverse studies that establish optimal parameters for different clinical scenarios. Our results suggest prioritizing PhNR amplitude over the PhNR/b-wave ratio when evaluating RGC function in myopic glaucoma patients using ERG. By identifying specific contexts where ERG measures are more or less useful, these findings support the broader integration of ERG as an objective functional diagnostic tool in clinical practice.

Previously, our group reported that an interocular difference in PhNR amplitude significantly correlates with the interocular difference in average RNFL thickness or GCIPL thickness, whereas no significant difference was observed between the mean sensitivity of VF tests and structural parameters [11]. The PhNR amplitude performed well in reflecting the inter-eye structure–function relationship in patients with early-stage glaucoma. However, this study shows that the diagnostic ability is relatively lower than that of OCT measurements of peripapillary RNFL thickness or GCIPL thickness, even in myopic patients. Therefore, clinicians may enhance the functional evaluation of glaucoma by performing PhNR amplitude or PhNR/b-wave ratio assessments along with structural evaluations of the RNFL or RGCs.

One of the limitations of this study is its relatively small sample size, which may affect the generalizability of the findings. Additionally, when evaluating the diagnostic ability for glaucoma, the control group consisted of glaucoma suspects rather than healthy individuals. This likely contributed to the relatively low diagnostic performance of PhNR amplitude for glaucoma compared to previous studies, where healthy controls were used. However, the inclusion of glaucoma suspects reflects the practical challenges of diagnosing glaucoma in clinical settings, where distinguishing glaucoma from glaucoma suspects is often more relevant than distinguishing glaucoma from healthy individuals. Although glaucoma is categorized as an age-related neurodegenerative disorder, the lack of significant age differences between the groups in this study may be due to the relatively small sample size and the inclusion of glaucoma suspects rather than healthy controls as the comparison group.

## 5. Conclusions

In conclusion, the diagnostic performance of the PhNR/b-wave ratio is not superior to that of PhNR amplitude in glaucoma patients. Our findings highlight the need for clinicians to consider the limitations of the PhNR/b-wave ratio in glaucoma patients with myopia due to the influence of AL on the b-wave. Further research is warranted to explore these relationships and enhance the diagnostic accuracy of electrophysiological tests in myopic glaucoma populations.

## Figures and Tables

**Table 1:** Demographics of patients with glaucoma or glaucoma suspects.

		Glaucoma Suspect (*n* = 19)	Glaucoma (*n* = 91)	*p*-Value
Age (years)		50.9 ± 12.1	51.1 ± 12.5	0.950
Male/Female		5/14	35/56	0.434
Central corneal thickness (µm)		548.7 ± 38.2	534.4 ± 50.9	0.219
Spherical equivalent (diopter)		−2.1 ± 3.4	−2.2 ± 2.8	0.846
Axial length (mm)		24.2 ± 1.6	24.7 ± 1.5	0.138
Spectral-domain OCT	Average RNFL thickness (µm)	90.9 ± 8.8	73.9 ± 10.0	**<0.001**
	Average GCIPL thickness (µm)	81.6 ± 4.2	70.3 ± 8.5	**<0.001**
	Minimum GCIPL thickness (µm)	77.9 ± 5.0	58.8 ± 13.3	**<0.001**
ERG	a-wave amplitude (µV)	32.6 ± 10.1	30.4 ± 8.0	0.313
	b-wave amplitude (µV)	112.4 ± 19.5	104.6 ± 25.6	0.213
	PhNR amplitude (µV)	35.9 ± 10.8	30.4 ± 10.3	**0.035**
	PhNR/b-wave ratio	0.32 ± 0.08	0.30 ± 0.10	0.370
Visual field 24-2	MD (dB)	−1.0 ± 1.4	−3.9 ± 3.6	**<0.001**
	PSD (dB)	1.6 ± 0.5	5.2 ± 3.4	**<0.001**

ERG, electroretinogram; GCIPL, ganglion cell inner plexiform layer; MD, mean deviation; OCT, optical coherence tomography; PhNR, photopic negative response; PSD, pattern standard deviation; RNFL, retinal nerve fiber layer. Statistically significant differences between the two groups (*p* < 0.05), uncovered by Student’s *t*-tests for continuous variables or chi-square tests for categorical variables, are indicated in bold.

**Table 2:** Comparison of patients based on myopic status.

	Non-Myopia (*n* = 42, AL < 24 mm)	Myopia (*n* = 68, AL ≥ 24 mm)	*p*-Value	Non-Myopia (*n* = 69, AL < 25 mm)	Myopia (*n* = 41, AL ≥ 25 mm)	*p*-Value
Age (years)	57.9 ± 9.1	46.9 ± 12.3	**<0.001**	54.9 ± 11.9	44.8 ± 10.3	**<0.001**
Male/Female	6/36	34/34	**<0.001**	25/62	15/8	**0.001**
Central corneal thickness (µm)	537.3 ± 34.6	535.4 ± 56.7	0.831	529.3 ± 40.1	547.2 ± 59.9	0.066
Spherical equivalent (diopter)	−0.2 ± 1.4	−3.4 ± 2.69	**<0.001**	−0.6 ± 1.4	−4.9 ± 2.8	**<0.001**
AL (mm)	23.2 ± 0.6	25.6 ± 1.1	**<0.001**	23.2 ± 086	26.2 ± 0.9	**<0.001**

AL, axial length. Statistically significant differences between the two groups (*p* < 0.05). Student’s *t*-tests for continuous variables or chi-square tests for categorical variables are indicated in bold.

**Table 3:** Electroretinograms and optical coherence tomography parameters in non-myopic and myopic patients.

	Parameters	Non-Myopia (*n* = 42, AL < 24 mm)	Myopia (*n*= 68, AL ≥ 24 mm)	*p*-Value	Non-Myopia (*n* = 69, AL < 25 mm)	Myopia (*n* = 41, AL ≥ 25 mm)	*p*-Value
Spectral-domain OCT	Average RNFL thickness (µm)	80.7 ± 10.6	74.5 ± 11.8	**0.006**	78.4 ± 11.5	74.3 ± 11.7	0.077
	Average GCIPL thickness (µm)	75.4 ± 8.2	70.4 ± 9.0	**0.004**	73.8 ± 8.8	69.8 ± 8.7	**0.023**
	Minimum GCIPL thickness (µm)	63.1 ± 15.7	61.5 ± 13.4	0.573	62.2 ± 15.5	62.0 ± 12.0	0.939
ERG	a-wave amplitude (µV)	31.59 ± 8.02	30.30 ± 7.85	0.438	30.85 ± 8.73	30.70 ± 7.85	0.929
	b-wave amplitude (µV)	110.34 ± 20.24	103.21 ± 26.91	0.142	108.72 ± 25.20	101.25 ± 23.47	0.127
	PhNR amplitude(µV)	32.28 ± 9.58	30.73 ± 11.17	0.457	30.64 ± 10.58	32.47 ± 10.60	0.383
	PhNR/b-wave ratio	0.30 ± 0.08	0.31 ± 0.11	0.590	0.29 ± 0.09	0.33 ± 0.12	**0.023**
Visual field 24-2	MD (dB)	−3.5 ± 3.9	−3.3 ± 3.2	0.784	−3.4 ± 3.9	−3.3 ± 2.7	0.917
	PSD (dB)	4.8 ± 3.9	4.4 ± 3.0	0.585	4.7 ± 3.6	4.4 ± 3.0	0.669

ERG, electroretinogram; GCIPL, ganglion cell inner plexiform layer; MD, mean deviation; OCT, optical coherence tomography; PhNR, photopic negative response; PSD, pattern standard deviation; RNFL, retinal nerve fiber layer. Statistically significant differences between the two groups (*p* < 0.05) using Student’s *t*-tests are indicated in bold.

**Table 4:** Correlation between electroretinogram parameters and axial length in patients with glaucoma or glaucoma suspects.

ERG Parameters	r	*p*-Value
a-wave amplitude (µV)	−0.108	0.261
b-wave amplitude (µV)	−0.230	**0.016**
PhNR amplitude (µV)	0.032	0.742
PhNR/b-wave ratio	0.239	**0.012**

ERG, electroretinogram; PhNR, photopic negative response. Statistically significant values (*p* < 0.05) according to Pearson’s correlations are highlighted in bold.

**Table 5:** Multivariate regression analysis of factors associated with the ERG parameters.

	Total				Non-Myopia (AL < 24 mm)				Myopia (AL ≥ 24 mm)			
Parameter	PhNR		PhNR/b-Wave		PhNR		PhNR/b-Wave		PhNR		PhNR/b-Wave	
	β Coefficient (95% CI)	*p*-Value	β Coefficient (95% CI)	*p*-Value	β Coefficient (95% CI)	*p*-Value	β Coefficient (95% CI)	*p*-Value	β Coefficient (95% CI)	*p*-Value	β Coefficient (95% CI)	*p*-Value
Age	−0.296 (−0.486~−0.107)	**0.003**	−0.001 (−0.03~0.001)	0.296	−0.271 (−0.591~0.049)	0.095	0.000 (−0.02~0.003)	0.802	−0.342 (−0.590~−0.094)	**0.008**	−0.002 (−0.004~0.000)	0.075
Gender	−3.212 (−7.778~1.353)	0.166	−0.045 (−0.091~0.000)	**0.049**	−9.182 (−17.445~−0.919)	**0.030**	−0.047 (−0.119~0.024)	0.189	−2.125 (−7.947~3.698)	0.469	−0.061 (−0.120~0.002)	**0.042**
AL	−0.718 (−2.446~1.011)	0.412	0.010 (−0.007~0.028)	0.229	−0.592 (−5.365~4.181)	0.803	0.012 (−0.029~0.054)	0.560	1.191 (−1.464~3.846)	0.373	0.028 (0.001~0.055)	**0.040**
Average GCIPL thickness	0.373 (0.156~0.589)	**0.001**	0.002 (0.000~0.004)	**0.036**	0.482 (0.145~0.823)	**0.006**	0.004 (0.001~0.007)	**0.004**	0.290 (0.007~0.573)	**0.045**	0.001 (−0.002~0.003)	0.681

AL, axial length; ERG, electroretinogram; GCIPL, ganglion cell inner plexiform layer; PhNR, photopic negative response. The variables that were statistically significant (*p* < 0.05) in the multivariate regression analysis are indicated in bold.

**Table 6:** Diagnostic performance of OCT and ERG parameters in distinguishing glaucoma patients from glaucoma suspects.

		Total				Non-Myopia (AL < 24 mm)				Myopia (AL ≥ 24 mm)			
		Mean AUC	*p*-Value	Sensitivity at Specificity > 80%	Cutoff	Mean AUC (95% CI)	*p*-Value	Sensitivity at Specificity > 80%	Cutoff	Mean AUC (95% CI)	*p*-Value	Sensitivity at Specificity > 80%	Cutoff
OCT	Average RNFL thickness (µm)	0.901 (0.827, 0.974)	**<0.001**	82.4	83.5	0.862 (0.757, 0.967)	**<0.001**	74.2	83.5	0.921 (0.853, 0.989)	**<0.001**	86.7	83.5
	Average GCIPL thickness (µm)	0.903 (0.842, 0.964)	**<0.001**	79.1	76.5	0.859 (0.757, 0.962)	**<0.001**	71.0	76.5	0.925 (0.866, 0.985)	**<0.001**	83.3	76.5
	Minimum GCIPL thickness (µm)	0.927 (0.878, 0.976)	**<0.001**	85.7	72.5	0.939 (0.875, 1.000)	**<0.001**	87.1	72.5	0.921 (0.861, 0.980)	**<0.001**	85.0	72.5
ERG	PhNR amplitude (µV)	0.661 (0.536, 0.786)	**0.028**	49.5	29.3	0.715 (0.566, 0.864)	**0.011**	58.1	29.3	0.633 (0.498, 0.767)	0.082	45.0	29.2
	PhNR/b-wave ratio (µV)	0.616 (0.490, 0.742)	0.114	38.5	0.26	0.696 (0.544, 0.849)	**0.021**	45.2	0.26	0.574 (0.440, 0.708)	0.332	35.0	0.26

AL, axial length; AUC, area under the receiver operating characteristics curve; ERG, electroretinogram; GCIPL, ganglion cell inner plexiform layer; OCT, optical coherence tomography; RNFL, retinal nerve fiber layer. Cutoff value at specificity > 80%. The variables that were statistically significant (*p* < 0.05) under the receiver operating characteristics curve are indicated in bold.

## Data Availability

The data presented in this study are available on request from the corresponding author. The data are not publicly available to maintain privacy.

## Abbreviations

The following abbreviations are used in this manuscript:

AUC	Area Under the Curve
AL	Axial Length
ERGs	Electroretinograms
GCIPL	Ganglion Cell Inner Plexiform Layer
MD	Mean Deviation
OCT	Optical Coherence Tomography
PhNR	Photopic Negative Response
RGCs	Retinal Ganglion Cells
RNFL	Retinal Nerve Fiber Layer
SD-OCT	Spectral-Domain Optical Coherence Tomography
VF	Visual Field
